# Scirrhus of the Common Bile Duct

**Published:** 1877-04

**Authors:** Norman Bridge


### SCIRRHUS OF THE COMMON BILE DUCT.

Reported by DR. NORMAN BRIDGE.

H. R. P., aged 65 years, American, a railroad conductor, a man of robust appearance; health generally good until the beginning of his last sickness. He was a good liver, and had, perhaps, as a consequence, an occasional diarrhœa. He had an erysipelas of the hand several years before death. An older brother died at 72 years of age, with cancer of the bile duct at the neck of the gall bladder.

His parents died of disorders not malignant, his mother from abscess of the lung following facial erysipelas.

Two and a half years before Mr. P.'s death he began to have spells of weakness and langor, at which times he would express himself as feeling that his "work was about done." These spells were of brief duration, and continued until one year before his death, when (spring of 1874) he caught cold and had some kind of a fever. He only partially recovered, was unfit for work, and lost flesh. He had pain frequently in the region of the liver and in the back. In August he went to Michigan for a visit of six weeks. While there he became deeply yellow with icterus—a condition that remained until his death. From November 1st, he was confined to his room.

He grew steadily weak and emaciated; anorexia was extreme; he would not eat—food appeased him and gave a feeling of distension of the stomach. There was no vomiting until within a week of his death, when it occurred frequently. A subjective feeling of enlargement of the liver had existed many months. A few weeks before death—and when the case fell into the hands of Dr. E. Ingals—there had appeared a considerable tumefaction in the liver, chiefly at the location of the gall bladder. By the localized prominence of the tumefaction, and by fluctuation, the mass was suspected to be an abscess. A day or two before death, however, a large quantity—one or two quarts—of bile and mucus was vomited, when the tumor was found to have disappeared. Death occurred May 3d, 1875.

The writer assisted Dr. Ingals in making a necropsy. The liver was somewhat enlarged and full of bile, that oozed from its cut surfaces. The gall-bladder was partially filled with bile having the usual appearance. A hard tumor, roundish, and not more than three-fourths of an inch in diameter, was attached to the common bile duct on its posterior aspect, and just above its entrance into the bowel. It appeared to have begun in the wall of the duct or the cellular tissue, just outside of it, and it had so encroached upon the lumen of the duct that it was with difficulty a small probe could be passed through the canal. The mass pressed slightly upon the duodenum, but it did not constitute an obstruction of that canal.

The other organs were normal. The tumor was found, on microscopic examination by Dr. Danforth, to be a scirrhus cancer.

Reported in June, 1875.

